# Gene Modification of Mesenchymal Stem Cells and Articular Chondrocytes to Enhance Chondrogenesis

**DOI:** 10.1155/2014/369528

**Published:** 2014-05-19

**Authors:** Saliya Gurusinghe, Padraig Strappe

**Affiliations:** ^1^School of Biomedical Sciences, Charles Sturt University, Wagga Wagga, NSW 2650, Australia; ^2^Graham Centre for Agricultural Innovation, Charles Sturt University, Wagga Wagga, NSW 2795, Australia

## Abstract

Current cell based treatment for articular cartilage and osteochondral defects are hampered by issues such as cellular dedifferentiation and hypertrophy of the resident or transplanted cells. The reduced expression of chondrogenic signalling molecules and transcription factors is a major contributing factor to changes in cell phenotype. Gene modification of chondrocytes may be one approach to redirect cells to their primary phenotype and recent advances in nonviral and viral gene delivery technologies have enabled the expression of these lost factors at high efficiency and specificity to regain chondrocyte function. This review focuses on the various candidate genes that encode signalling molecules and transcription factors that are specific for the enhancement of the chondrogenic phenotype and also how epigenetic regulators of chondrogenesis in the form of microRNA may also play an important role.

## 1. Introduction

The self-healing capacity of articular cartilage is minimal when damaged, due to the avascular nature of the articular joint surface. This reduces the potential for wound healing progenitor cells to access the site of injury for regeneration of the articular tissue [1]. The current cell based treatment methods for cartilage repair are based on the migration of bone marrow derived stem cells to the site of injury and include arthroscopic abrasion, subchondral drilling, and microfracture [2]. Exogenous cell based tissue engineering applications to induce cartilage formation include periosteum and perichondrium grafts and implantation of culture expanded autologous chondrocytes or implantation of culture expanded, bone marrow derived mesenchymal stem cells (MSCs) [3, 4]. Both embryonic stem cells (ES) and adult somatic stem cells of mesenchymal origin have been used to form cartilage, both* in vitro* and* in vivo*. ES cells have been shown to differentiate into chondrocytes in a two-step process, where initially the stem cells change their phenotype to chondrogenic progenitors, followed by differentiation of these progenitor cells into chondrocytes.* In vitro* differentiation of these stem cells is highly efficient when combined with a three-dimensional microenvironment, with the addition of growth factors to enhance differentiation [5].

Both MSCs and chondrocytes are permissive to gene transfer and as such are excellent candidates for gene modification to enhance their chondrogenic phenotype and promote proliferation, avoiding detrimental cellular dedifferentiation, and senescence [6]. Gene delivery to MSCs and chondrocytes has been carried out to stimulate anabolic pathways lost through dedifferentiation by the expression of growth factors and transcription factors [6], to repress the activation of catabolic pathways [7], or to address a combination of these factors [8].

The introduction of foreign DNA encoding a gene of interest directly into a living cell results in the degradation of the naked DNA and therefore requires an efficient carrier for its delivery to the cell nucleus for gene transcription and subsequent protein expression [9]. Physical barriers such as the cellular membrane prevent the entry of the nucleic acids reducing the efficiency of transfection. DNA taken into the cells by endocytosis exists within endosomes which may then transform into digestive lysosomes resulting in nuclease activity to break down the DNA. To overcome endosomal entrapment, DNA in the form of a plasmid can be associated with a cationic liposome for cellular entry [10]. However, if long-term transgene expression is required, viral based gene delivery techniques should be considered.

## 2. Nonviral Vectors for Gene Delivery

A major advantage in using nonviral gene delivery techniques is the lack of transgene integration in the host genome. However, the delivery methods themselves are challenging due to the high level of cytotoxicity seen during plasmid DNA ransfections with liposome based transfection systems [11]. Increasing the transfection efficiency is dependent on the careful titration of the liposomal transfection reagent to minimize cytotoxicity while maintaining high DNA uptake and the optimisation of the ratio of DNA to liposomal agent [12]. Current nonviral gene delivery strategies incorporate the use of biocompatible polymeric scaffolds to facilitate slow release of plasmid DNA which can enhance transfection efficiency and together with three-dimensional cell culture conditions supporting the chondrogenic phenotype in transfected cells [13–16].

## 3. Viral Vectors for Gene Delivery

Efficient transgene delivery into MSCs and articular chondrocytes has been achieved using a variety of viral based vectors including adenovirus, recombinant adeno associated virus (rAAV), retrovirus, and lentiviral vectors. Each of these viral vectors has inherent advantages and disadvantages associated with their function [17]. The most widely used viral vectors for cartilage repair are adenoviral vectors which are advantageous due to their high transduction efficiency, broad cell tropism, and the reduced immunological response particularly at avascular synovial joints, reducing the probability of gene silencing [14, 18]. However, silencing of adenoviral vector delivered transgenes has been observed in* in vitro* models of chondrogenesis with high doses of viral vector [19, 20]. On the contrary, less immunogenic rAAV vectors have been identified as ideal candidates for viral gene delivery due to their nonpathogenic nature and are made more desirable due to the maintenance of transgene expression in an episomal form reducing the risks associated with insertional mutagenesis [17].

Retroviral vectors predominantly based on the murine leukaemia virus (MLV) transduce dividing cells and also integrate transgenes into the host genome which allow continued transgene expression but can potentially lead to insertional mutagenesis as experienced in an early human trial [21]. Similarly, lentiviral vectors also integrate into the host genome for long-term transgene expression [22, 23]. In contrast to retroviral vectors, lentiviral vectors are capable of delivering genes to both dividing and nondividing cells and are predominantly based on human immunodeficiency virus type 1, although other lentiviral vectors based on simian immunodeficiency virus, feline immunodeficiency virus, and equine infectious anaemia virus have been described. Lentiviral vectors also possess the risk of insertional mutagenesis [11].

Pseudotyping lentiviral vectors with a heterologous envelope such as the commonly used glycoprotein derived from vesicular stomatitis virus (VSV-G) can provide structural integrity to the virus particle and also enhance tropism for a wide variety of cell types [24]. Numerous other envelopes have been used to pseudotype lentiviral vectors, often in an attempt to selectively restrict tropism to a certain cell type [25]. Important biosafety features are also engineered into the lentiviral vector to minimize the probability of the assembly of recombinant competent HIV-1; the essential structural and functional proteins of the virus have been separated into individual expression plasmids. Further advances in lentiviral technology have given rise to expression systems with minimal viral genome sequences while maintaining transduction efficiency [26] and enhancing biosafety [27].

## 4. Reporter Gene Expression in MSCs and Articular Chondrocytes 

A distinct advantage of using a reporter gene primarily in the form of a fluorescent protein is the relatively easy quantification of transduction efficiency and visualisation of transgene localisation. Delivery of the commonly used green fluorescence protein (GFP) reporter gene has been extensively used to document and track the efficiency of gene delivery by both viral and nonviral systems to MSCs and chondrocytes.

Viral based gene delivery to adipose derived multipotent stem cells has been efficiently performed using adenoviral, retroviral, and lentiviral vectors expressing enhanced green fluorescent protein (eGFP) [28]. All three delivery methods resulted in high transgene expression with the highest efficiency associated with lentiviral transduced cells. Differentiation of the multipotent cells subsequent to transduction showed that adenoviral transductions at higher titres result in cytotoxicity. This toxicity was also demonstrated in a later study using MSCs where higher viral titres resulted in a reduction in cell proliferation [19, 20]. However, retroviral and lentiviral vectors successfully transduced these cells avoiding cell toxicity with transgenes maintaining expression over a 100 day period.

The efficiency of gene delivery is also relative to the cell type used. For example it has been shown that viral gene delivery to primary human chondrocytes could be carried out successfully with the use of an AAV vector to deliver GFP which was expressed in these cells at a high efficiency of 93.7% at 7 days after transduction and gene expression was maintained for up to 28 days after transduction [29]. Importantly it was observed that the viral transduction of these cells did not alter their capacity to proliferate or maintain the chondrogenic phenotype. In order to gain control of GFP transduction, Ulrich-Vinther et al. [30] used light activated gene transduction in an AAV system and obtained a transduction efficiency of approximately 50% in cultured rabbit chondrocytes. Although comparatively lower transduction efficiency was observed, there was a high degree of tissue specificity with this system.

In contrast, GFP delivered via AAV vectors to MSCs have shown a maximum transduction efficiency of 65% [31]. Importantly, it was shown that the exogenous GFP expression in these cells did not affect their capacity to differentiate into end stage cell types such as chondrocytes, osteocytes, and adipocytes suggesting the appropriateness of the viral based gene delivery methods for therapeutic use due to their minimal impact on normal cellular function.

## 5. Overexpression of Transcription Factors for Chondrogenic Enhancement

Enhancement of the chondrogenic phenotype can be achieved by ectopic expression of transcription factors to regulate subsequent expression of their target genes which are often lost due to cellular dedifferentiation or in pathological conditions such as osteoarthritis. Representative studies related to the use of these factors are listed in Table 1. Of particular importance in cartilage tissue engineering is the induction of the major cartilage matrix proteins such as collagen II and aggrecan [6, 22, 32]. Several methods of enhancing collagen II expression have been reported and the main transcription factor recognised for this purpose is Sox9 as it directly enhances collagen II expression by transcriptionally activating the enhancer region of the* col2a1* gene in both differentiating MSCs and chondrocytes [6].

### 5.1. SMAD3

Smad3 is an important downstream transcription factor in the TGF*β* receptor mediated chondrogenic induction pathway and important in the activation of Sox9 transcription. Adenoviral transduction of human MSCs with Smad3 cDNA resulted in strong upregulation of extracellular matrix protein secretion [33]. The association of phosphorylated Smad3 with recombinant Sox9 has been shown to recognize the enhancer region of the* col2a1* gene encoding collagen II directly influencing its expression [34].

### 5.2. Sox9

Efficient ectopic expression of Sox9 has been demonstrated by nonviral transfection of Sox9 cDNA into mouse MSCs* in vitro* by lipofection [32]. Subsequently these cells were transplanted onto athymic mice for a period of 4 weeks, after which histological staining showed glycosaminoglycan production. Interestingly, although the transfection efficiency of the Sox9 gene was lower than that of viral based systems, it was noted that the overall expression of Sox9 was capable of enhancing the chondrogenic phenotype.

The overexpression of* Sox9* has also been achieved with adenovirus, rAAV [35–37], retrovirus [38], and lentivirus [6]. In general, these viral based gene delivery systems were capable of delivering the* Sox9* gene efficiently for both* in vitro* and* in vivo* applications. A comparative study into the use of these viruses to deliver* Sox9* to human osteoarthritic chondrocytes* in vitro* showed reduced dedifferentiated phenotype and the formation of hyaline like cartilage. At a transduction efficiency of 85%, lentiviral transduction was identified as an appropriate vector system for* Sox9* delivery for translational applications such as autologous chondrocyte transplantation [6].

The delivery of sox9 by AAV vectors has also been shown to be efficient in explant cultures of human cartilage, with enhanced expression of extracellular matrix components [35].* In vitro* AAV mediated* Sox9* overexpression in human bone marrow derived mesenchymal stem cells resulted in a significant reduction in hypertrophy markers. Nonviral delivery of* Sox9* plasmid DNA has been shown to enhance chondrogenesis of human MSCs when codelivered with siRNA to the osteogenic transcription factor Cbfa-1 [39].* Sox9* delivery by adenoviral vector into rabbit MSCs has shown that the ectopic expression was maintained when transduced cells were implanted into osteochondral defects in rabbits resulting in successful integration of the graft tissue [40]. A tetracycline inducible* Sox9* transfection system using a biodegradable scaffold for chondrogenic stimulation has also been used to enhance the phenotype of rat chondrocytes. This combined approach may bring* Sox9* mediated gene therapy closer to clinical application [41].

### 5.3. Barx2

The homeobox transcription factor Barx2 has also been recognised as a regulator of chondrogenesis during embryonic development.* In vitro* retroviral transduction of mouse embryonic MSCs with the transcription factor* Barx2* showed an increase in cell aggregation prior to chondrogenic differentiation indicating its capacity to enhance cell-cell interaction [42]. Retroviral vector overexpression of the gene along with Sox9 resulted in an increase in collagen II production due to the enhancement of* col2a1* through* Barx2* interaction with Sox9. This study may indicate the potential for the cotransduction of this novel transcription factor with* Sox9* for enhancing the expression of collagen II in adult stem cells or dedifferentiated chondrocytes.

## 6. Stimulatory Growth Factors for the Enhancement of Chondrogenesis

Growth and differentiation factors are important in chondrogenic differentiation of MSCs and in the maintenance of the chondrogenic phenotype. Particular members of the TGF*β* superfamily of growth factors are therefore potential candidates for gene therapy applications.

### 6.1. BMP-2

Bone morphogenetic protein-2 (BMP-2) has mainly been used to enhance osteogenesis and facilitate repair of critical size defects in bone; however, some studies have also shown increased chondrogenesis upon ectopic expression of this factor [43]. Adenoviral transduction of MSCs with* BMP-2* resulted in higher chondrogenic potential, evidenced by both increased levels of cell proliferation and collagen II matrix protein secretion. The degree of chondrogenic differentiation was observed to be highly influenced by the cell type. Perichondrium derived cells demonstrated the highest chondrogenic capacity followed by bone marrow derived stem cells and fat derived stem cells. Importantly the formation of fibro cartilage was observed moderately in bone marrow derived stem cells and significantly higher in fat derived stem cells highlighting the importance in the choice of cell type for genetic modification in translational clinical applications. In agreement with this study Pan et al. [44] showed that* BMP-2* enhanced chondrogenesis through plasmid DNA delivery of* BMP-2* cDNA which resulted in an increase in Sox9 expression, the major transcription factor in the regulation of chondrogenesis. A recent study has also demonstrated the same effect and has shown that BMP-2 expression is lowered in Sox9 enhanced chondrogenesis [45]. This finding is highly significant when considering the application of BMP-2 for chondrogenic enhancement in a clinical setting, as constitutive expression of BMP-2 can lead to chondrocyte hypertrophy, followed by osteogenic differentiation.

### 6.2. BMP-7

Interestingly, bone morphogenetic protein-7 (*BMP-7*) overexpression by adenoviral transduction in equine chondrocytes has shown that articular cartilage matrix generation occurs earlier than untransduced control cells when transplanted* in vivo* [18]. At 4 weeks after transplantation, a high wound healing capacity was observed in defects treated with the* BMP-7* transduced cells when compared to controls. However, at 8 months the difference in matrix formation between the BMP-7 treated and control cultures was not significant, indicating that BMP-7 might initially accelerate wound healing process. Furthermore, the compressibility of the defects treated with* BMP-7* transduced chondrocytes showed a 2- to 10-fold reduction in weight bearing capacity, suggesting the need to activate alternative chondrogenic pathways to enhance mechanical properties. Transgene silencing associated with adenoviral transduction was not observed and immunological response to the virus was significantly low in articular joints, supporting the use of adenovirus for efficient gene transfer and expression. Gavenis et al. [46] showed that human articular chondrocytes could also be cultivated in polylactic microspheres loaded with* BMP-7*, enabling long-term culture expansion, while retaining the hyaline cartilage phenotype of the cells and high resistance to dedifferentiation.

### 6.3. TGF*β*-1

MSCs modified with the TGF*β*-1 signalling molecule have shown greater potential for chondrogenic differentiation when compared to unmodified cells.* TGF*β*-1* when expressed using an adenoviral delivery system was capable of enhancing chondrogenesis in MSCs secreting approximately 5 ng/mL of the peptide into the culture media in the modified cells when compared to unmodified cells that required 10 ng–24 ng of exogenous TGF*β*-1 in the protein form to achieve the same effect [47]. The results suggest that constant expression of* TGF*β*-1* through gene modification was more effective than intermittent addition of growth factor proteins with short half-lives. In an attempt to develop an* in vitro* model of a rabbit osteochondral junction with MSCs differentiating to chondrocytes in one layer and osteocytes in a second layer, Chen et al. [48] showed that chondrogenic induction of MSCs could be achieved by immobilisation of plasmid DNA expressing TGF*β*-1 in a chitosan based scaffold in a bilayered system.

### 6.4. TGF*β*-3

TGF*β*-3 is also an important stimulatory molecule that drives chondrogenesis by activating the Smad signalling pathway. This factor is mainly used in the recombinant protein form when administered in cell culture [49, 50]. However, it has also been delivered by viral vector to drive chondrogenesis.* TGF*β**-*3* has been expressed via lentiviral vectors immobilised onto poly(*ε*-caprolactone) (PCL) woven scaffolds to transduce human bone marrow derived MSCs [51]. Transduced MSCs undergoing chondrogenic differentiation demonstrated similar levels of extracellular matrix production when compared to MSCs that received recombinant TGF*β*-3 protein.* TGF*β*-3* has also been delivered to adipose derived stem cells (ADSCs) using a baculoviral vector [52], in which TGF*β*-3 expressing cells displayed enhanced collagen II and matrix protein expression improved biomechanical properties.

### 6.5. IGF-1

Insulin-like growth factor-1 (IGF-1) enhances cell proliferation and differentiation in articular chondrocytes during growth plate development [53]. Nonviral delivery of* IGF-1* into rabbit articular chondrocytes has shown enhanced glycosaminoglycan production increasing over time [54]. In contrast, adenoviral expression of* IGF-1* in human MSCs did not enhance chondrogenesis and inhibited collagen II expression [47]. This observation would suggest specificity in* IGF-1* function in different cell types.* IGF-1* plasmid DNA immobilised onto collagen II-glycosaminoglycan scaffolds has been delivered to canine articular chondrocytes to facilitate slow release of the plasmid DNA at sustained therapeutic levels [55]. The resulting tissue formation showed a greater volume, elevated glycosaminoglycan production, and matrix specific collagen II synthesis in the scaffold. The transfection efficiency was further optimised by the incorporation of cationic gelatine scaffolds in a later study [56].* IGF-1* plasmid DNA transfected rabbit articular chondrocytes have been used to treat* in vivo* cartilage defects in combination with fibroblast growth factor-2 (*FGF-2*) resulting in articular cartilage regeneration with low collagen I expression [57].

### 6.6. FGF-2

Fibroblast growth factor-2 (*FGF-2*) has been delivered to rabbit chondrocytes by transfection of plasmid DNA and the transplantation of the transfected cells demonstrated enhanced collagen II expression. Importantly the expression of fibrocartilage marker collagen I was detected; however, the level of expression was lower than that of the control [58]. The effect of rAAV delivered* FGF-2* has also been studied in human MSCs demonstrating strong chondrogenic differentiation potential with enhanced cartilage matrix production and exhibiting low fibrocartilage and osteogenic markers [59].

## 7. Delivery of Multiple Factors to Enhance Chondrogenesis

Chondrogenic differentiation of MSCs into chondrocytes can often result in the formation of cartilage tissue rich in predominantly fibrous collagen I fibres, referred to as fibrocartilage. A number of studies have used a combination of genes to address cellular hypertrophy and dedifferentiation.

### 7.1. TGF*β*-3 and Col1-shRNA

To enhance chondrogenic differentiation in synovium derived MSCs by growth factor and short hairpin RNA expression, Zhang et al. [8] used bicistronic lentiviral vector delivery of the growth factor* TGF*β*-3* as well as a short hairpin RNA (shRNA) targeting the* col1* gene. This resulted in enhanced expression of the type II collagen and a significant reduction in collagen I expression after culturing transduced cells encapsulated in an alginate hydrogel for subsequent* in vivo* transplantation. This* ex vivo* strategy was used to eliminate the carryover of immunogenic viral components by subsequent passaging to allow their dilution in culture.

### 7.2. BMP-2, TGF*β*-1, and IGF-1

Individual treatments of* BMP-2* [43],* TGF*β*-1*, and* IGF-1* [47] have been shown to enhance chondrogenesis by activating the extracellular matrix protein synthesis pathways. Adenoviral cotransduction of human bone marrow derived MSCs* in vitro* with insulin-like growth factor (*IGF-1*) and* TGF*β*-1* or* BMP-2* has been shown to have synergistic effects in terms of chondrogenic enhancement with increased collagen II expression [19, 20]. Importantly the use of lower viral doses of each factor was shown to be critical for higher expression as adenoviral transductions at high viral doses were shown to inhibit chondrogenesis. However, this study demonstrated a high level of collagen X expression in transduced cells.

### 7.3. IGF-1 and FGF-2

The synergistic effects of* IGF-1* and* FGF-2* in chondrogenic differentiation have also been shown in ovine adipose derived stem cells with adenovirus transduced cells demonstrating very low expression of fibrocartilage marker collagen I or hypertrophic marker collagen X [60].

### 7.4. Sox9 and BMP-2

*Sox9* and* BMP-2* have also been delivered in a bicistronic nonviral plasmid vector into dedifferentiated human chondrocytes expressing a weak chondrogenic phenotype [61]. Overexpression of the two genes resulted in a synergistic effect with rapid redifferentiation of the chondrocytes with high levels of extracellular matrix component expression.

### 7.5. Klf4, c-Myc, and Sox9

As a successful expression system, retroviral transduction has been used to deliver transcription factors important in the reprogramming of somatic cells to obtain stem cell like phenotypes. Previously Takahashi and Yamanaka [62] demonstrated that a specific group of transcription factors (*Oct4*,* Klf4*,* C-Myc*, and* Sox2*) were capable of reprogramming mouse dermal fibroblast to obtain a pluripotent stem cell phenotype.

Combining these transcription factors along with chondrogenic transcription factor* Sox9*, Outani et al. [22] were able to differentiate mouse dermal fibroblasts directly into chondrocytes, by passing the induced pluripotent stem cell state. Of these transcription factors, the overexpression of* Klf4* and* c-Myc* with* Sox9* yielded the highest efficiency in chondrogenic differentiation resulting in extracellular matrix secretion, with physical and histological properties as those of articular cartilage. This result has also been shown in the direct differentiation of human dermal fibroblasts to chondrocytes with the same factors [63]. The codelivery of one or more chondrogenic factors along with potential repressors of hypertrophy or dedifferentiation may promote the maintenance of the chondrogenic phenotype.

## 8. Telomerase Activity for Immortalization of MSCs and Chondrocytes

A reduction in the rate of proliferation in chondrocytes, as with most somatic cell types, is greatly affected by telomere shortening as these cells divide, affecting the total number of healthy cells available for chondrocyte transplantation. As a comparatively novel approach for obtaining sufficient human chondrocyte numbers by culture expansion, retroviral vector mediated forced expression of the human telomerase reverse transcriptase (*hTeRT*) gene has been performed [64]. The cellular immortalization resulted in a greater expansion of osteoarthritic chondrocytes which under normal physiological conditions would become senescent at lower passages. The immortalized chondrocytes demonstrated accelerated proliferation and comparable collagen II expression to normal chondrocytes as well as reduced collagen I expression. Telomerase overexpression has also been used to immortalize somatic multipotent stem cells due to their reduced capacity to self-renew in comparison to embryonic stem cells. In order to immortalize human placenta derived mesenchymal progenitor cells,* hTeRT* has been coexpressed with* Bmi-1* oncogene with a lentiviral vector to prolong the lifespan of the immortalized cells [65]. In order to control the expression of exogenous* hTeRT* expression in human MSCs, a Tet-On inducible system has been used previously which showed that the transduced cells ceased proliferation when doxycycline was removed [66, 67]. The immortalization of human bone marrow derived MSCs with* hTerRT* has also shown that the chondrogenic differentiation capacity of MSCs is retained with its forced expression [68, 69].

## 9. MicroRNA Expression to Enhance Chondrogenic Differentiation

MicroRNAs (MiRNAs) are a group of noncoding short RNA molecules that bind to the 3′ untranslated region of target mRNA, resulting in the repression of gene translation and can be delivered to cells by means of plasmid DNA encoding the parental form of the microRNA which after enzyme cleavage yields the mature and functional microRNA [70]. A list of representative studies related to the overexpression of selecting miRNAs for the enhancement of chondrogenesis has been listed in Table 2. The regulatory properties of microRNA in chondrogenesis have been demonstrated by Lin et al. [71] where prechondrogenic cell lines undergoing chondrogenic induction with BMP-2 were repressed by the forced expression of miR-199a delivered by a nonviral gene vector. This study noted the decreased expression of the cartilage specific matrix proteins collagen II and cartilage oligomeric matrix protein (COMP) and also a repression in the transcription factor Sox9 indicating a regulatory role for miR-199a in chondrogenesis. It was suggested that the repression of chondrogenesis was associated with the inhibition of* Smad1* mRNA by direct binding to the 3′ untranslated region of the transcript. In contrast, it has been found that Sox9 driven chondrogenesis could be enhanced by the expression of the microRNA-675 (miR-675). This mature microRNA is formed by the cleavage of the parental microRNA transcript H19, and its overexpression has been shown to result in Sox9 enhanced* col2a1* expression* in vitro* but not in Sox9 induced expression of* Col9a1* [72]. The target of this microRNA has been hypothesised to be a repressor of collagen II, derepressing the expression of* col2a1*. The target could potentially be histone deacetylase I or II, of which inhibition resulted in the elevated expression of collagen II in arthritic chondrocytes [73]. The inability of miR-675 and histone deacetylase I and II to influence expression of* col9a1* may suggest a link in their regulatory mechanism.

The repression of Sox9 has been demonstrated with the forced expression of microRNA-145 (miR-145) in the C3H10T1/2 cell line* in vitro*.* miR-145* targets Sox9 protein directly resulting in a decrease in cartilage matrix proteins [74]. Inhibition of this microRNA was also shown to result in elevated expression of Sox9-dependant downstream targets, miR-675, type II collagen, aggrecan, and COMP resulting in an enhanced chondrogenic phenotype. miR-145 forced expression in normal human chondrocytes has demonstrated the same trend with strong knockdown of Sox9 protein levels [75]. However, the expression of this microRNA in chondrocytes from osteoarthritic cartilage remains to be seen. A strong inhibitor of the major transcription factor Sox9 such as miR-145 therefore presents a very attractive target for gene therapy applications if elevated levels are detected under osteoarthritic conditions.

MicroRNAs have also been associated with the prevention of senescence and apoptosis in chondrocytes. MicroRNA-21 (miR-21) has been shown to enhance cell proliferation in rat articular chondrocytes when delivered* in vitro* [76]. The maintenance of cell proliferation while retaining the capacity of chondrocytes to express matrix proteins such as collagen II may be regulated by miR-21 due to its repression of tumour suppressor genes.

Highly specific to cartilage tissue, microRNA-140 (miR-140) has been studied extensively for its ability to inhibit the expression of chemokine (C-X-C Motif) ligand 12 (CXCL12) and A disintegrin and metalloproteinase with thrombospondin motifs 5 (ADAMTS-5) that lead to articular cartilage degradation in osteoarthritis [77]. MicroRNA array analyses have shown that miR-140 represses CXCL12 which leads to chondrogenic hypertrophy [78]. However, the physiological effects on articular chondrocytes resulting from miR-140 repression have not been evaluated.

*ADAMTS-5* is a cleavage enzyme that degrades the major cartilage protein aggrecan and is highly expressed in osteoarthritic cartilage [79]. It has been shown that miR-140 null mice demonstrate a highly osteoarthritic pathology at the knee joints due to derepression of* ADAMTS-5*.* In vivo* expression of miR-140 in transgenic mice with the transcript driven by the* col2a1* promoter has shown specific articular cartilage matrix expression and resistance to arthritis [80].

The potential for microRNAs to control the chondrogenic process may have future translational application; in particular the overexpression of candidate miRNAs such as miR-140, miR-21, and miR-675 may enhance the chondrogenic potential of both MSCs and culture expanded chondrocytes.

## 10. Gene Therapy in Human Trials

The first and only gene therapy human trial for treating an osteochondral defect has been the use of the interleukin-1 receptor antagonist* IL-1Ra against inflammation caused during rheumatoid arthritis* [7], where synovial fibroblasts were transduced with a retrovirus carrying* IL-1Ra*. Intra-articular injections of these cells resulted in significantly lower inflammation relative to the control group and importantly no adverse effects were observed in patients undergoing treatment. At the time of writing only one clinical trial involving gene therapy to treat arthritis had been listed, with allogeneic human chondrocytes expressing* TGF*β*-1* to be used for the treatment of cartilage defects (http://www.clinicaltrials.gov/). Future advancement in gene therapy techniques leading to clinical trials may require highly efficient gene transfer systems, such as self-complimentary AAV vectors (scAAV), providing more efficient gene transduction compared to traditional single stranded vectors with high levels of safety. Recent work demonstrates their efficiency* in vivo* [81].

## 11. Summary

A wide variety of transgenes and gene delivery vectors have been used to enhance chondrogenic differentiation in adult stem cells and to reduce dedifferentiation and hypertrophy in articular chondrocytes. The current challenge for the clinical applications of gene modified cells is enhancing the safety of gene delivery vectors while maintaining therapeutic levels of transgene expression. Advances in higher efficiency, scaffold employing nonviral gene delivery systems, and enhanced safety features in viral based gene delivery systems have shown great potential for clinical application of such technologies.

## Figures and Tables

**Table 1:** Candidate genes to enhance chondrogenesis in MSCs and dedifferentiated chondrocytes.

Overexpressed factor	Cell type	Outcome	References
TGF*β*-1	Human bone marrow derived MSCs, rabbit bone marrow MSCs	Enhanced chondrogenic differentiation through Smad signalling, downregulation of sonic hedgehog signalling	[19, 20] [48, 82]
BMP-2	Human bone marrow derived MSCs, perichondrial/periosteal cells, and adipose derived stem cells	Enhanced chondrogenesis driven by Sox9 activated collagen II and aggrecan synthesis, accelerated chondrocyte hypertrophy	[43, 44] [19, 20] [45]
TGF*β*-3	Rat adipose derived stem cells, porcine synovium derived MSCs	Enhanced chondrogenic potential and high resistance to fibro cartilage formation	[8, 83, 84]
BMP-7	Equine chondrocytes	Early onset of cartilage specific matrix synthesis, resistance to chondrocyte hypertrophy	[18, 46]
IGF-1	Rabbit articular chondrocytes, human bone marrow derived MSCs	Chondrocyte proliferation, enhanced wound healing potential in osteochondral defects, enhanced extracellular matrix synthesis	[53–55] [19, 20] [56, 57, 60]
FGF-2	Rabbit articular chondrocytes	High rate of cell proliferation, enhanced collagen II expression and formation of fibrocartilage	[58–60]
SMAD-3	Human MSCs	Activation of Sox9 resulting in collagen II over expression	[33, 34]
SOX-9	Mouse MSCs, human chondrocytes (normal and osteoarthritic), human bone marrow derived MSCs	Binding to *col2a1* enhancer increases collagen II synthesis	[6, 32, 35, 36, 39–41, 65]
BARX-2	Mouse embryonic MSCs	Cell aggregation, association with Sox9 to bind and enhance collagen II expression	[42]
Klf4/c-Myc	Mouse dermal fibroblast, human dermal fibroblasts	Direct differentiation of dermal fibroblast to chondrocytes with high expression of articular chondrocyte phenotype	[22, 63]
hTeRT	Human chondrocytes, human placenta derived MSCs, human bone marrow derived MSCs,	Enhanced cell proliferation while maintaining the capacity for chondrogenic differentiation	[64, 66–69]
IL-1Ra	Human synovial fibroblast	Reduction in inflammation in joint capsule	[7]

**Table 2:** Candidate miRNAs to enhance chondrogenesis in MSCs and dedifferentiated chondrocytes.

MicroRNA	Cell type used	Result	Reference
miR-199a	C3H10T1/2 stem cells	Knockdown of Smad1	[71]
miR-675	Human articular chondrocytes	Enhancement of Collagen II synthesis	[72]
*miR-145 *	C3H10T1/2 stem cells	Knockdown of Sox9	[74, 75]
*miR-21 *	Rat articular chondrocytes	Enhancement of chondrocyte proliferation	[76]
miR-140	C3H10T1/2 stem cells	Knockdown of ADAMTS-5 and CXCL12	[77, 78]
